# Systems that evaluate international equivalency in health-related professions: a scoping review with a focus on Canada

**DOI:** 10.1186/s12960-023-00864-y

**Published:** 2023-10-06

**Authors:** Mark Lafave, Yasaman Amannejad, Ulkar Mammadova, Breda Eubank

**Affiliations:** Mount Royal University, 4825 Mount Royal Gate SW, Calgary, AB T3E 6K6 Canada

**Keywords:** Competence, Professional, Health workforce, Immigration, Equivalency

## Abstract

Health workforce planning has become a significant global problem considering there are estimates of an 18 million healthcare provider shortfall by 2030. There are two mechanisms to address healthcare worker shortages: (1) domestic education of those professions and (2) integration of internationally educated health professionals. Integration of internationally educated health professionals into the Canadian healthcare system requires: (1) reductions in systemic and administrative barriers and (2) development, testing, and implementation of credential equivalency recognition systems. The goal of this scoping review was to identify systems that are employed to determine credential equivalency, with a focus on Canada. The scoping review was carried by employing: (1) a systematic literature search (9) and (2) a website and grey literature Google search of professional governing bodies from a selection of medical/allied healthcare professions, but also other non-medical professions, such as law, engineering and accounting. Seven databases were searched to identify relevant sources: MEDLINE, CINAHL Plus with Full Text, PsycINFO, SPORT Discus, Academic Search Complete, Business Source Complete, and SCOPUS. The search strategy combined keyword, text terms, and medical subject headings (MeSH) and was carried out with the help of a health sciences librarian. Seven articles were included in the final manuscript review from the following professions: nursing; psychology; engineering; pharmacy; and multiple health professions. Twenty-four health-related professional governing body websites were hand searched to determine systems to evaluate international equivalency. There were many systems employed to determine equivalency, but there were no systems that were automated or that employed machine-learning or artificial intelligence to guide the evaluation process.

**Supplementary Information:**

The online version contains supplementary material available at 10.1186/s12960-023-00864-y.

## Background

Health workforce planning has become a significant global problem considering there are estimates of an 18 million healthcare provider shortfall by 2030 [1]. These projections were made prior to the COVID-19 pandemic and thus, one could speculate those shortages are even greater given the attrition rates associated with managing the impact of COVID-19 burnout on healthcare workers [2]. Generally speaking, there are two mechanisms to address healthcare worker shortages: (1) domestic education of those professions and (2) integration of internationally educated health professionals. Asamani et al. [3] and Ono et al. [4] identified planning models that could help predict the projected health resource need of societies/countries based on demographics, healthcare utilization rates, and disease states that require healthcare services. To determine if needs were being met, Scheffler et al. performed a gap analysis by comparing healthcare worker output from domestic educational sources internally and migrant workers coming from external educational or healthcare systems. They found that “the Western Pacific region will have a very strong economic demand for health workers that will not be met by growth in domestic supply and is, therefore, likely to exert a strong unmet demand pressure on the supply of health workers from low- and lower-middle-income countries.” ([1], p. 6) Consequently, systems that facilitate bilateral movement between countries are more likely to address these needs more efficiently.

Integration of internationally educated health professionals into the Canadian healthcare system requires: (1) reductions in systemic and administrative barriers and (2) development, testing, and implementation of credential equivalency recognition systems [5]. Unfortunately, Canada, has yet to date, met any of the key milestones the World Health Organization as set out to address health workforce mobility [6]. The bottom line is that health workforce planning is extremely complex with many challenges to overcome.

Two important international organizations have dedicated resources to health workforce planning globally: the World Health Organization (WHO) [7] and the Organization for Economic Cooperation and Development (OECD) [8]. As referenced above, the WHO published the “Global strategy on human resources for health: Workforce 2030” and made a number of recommendations for member states [9]. The report suggests that past strategies addressing persistent workforce challenges be re-evaluated to adopt a paradigm shift that leverages existing skillsets and highlights the necessity of labor mobility. To ensure quality control, the report also advises “governments to collaborate with professional councils and other regulatory authorities to adopt regulation that takes into account transparency, accountability, proportionality, consistency, and that is targeted to the population’s needs.” [9] The recommendation proposes working with professional regulators to ensure that the healthcare workforce is competent, experienced and adhering to local standards. Specifically, “to avoid potential conflicts of interest, governments, professional councils and associations should create *appropriate mechanisms* to separate their role as guarantor of the quality of practice for the benefit of public health objectives from that of representing the interests of their members.” [9] For the purposes of this paper, we would characterize “appropriate mechanisms” as credential equivalency recognition systems that scrutinize international professional equivalencies, and do not have any real, perceived, or potential conflicts of interest with their respective government or professional associations.

A scoping review method was chosen for this study due to the unique nature of the research question and data we are seeking to extract from traditional literature and the grey literature. Moreover, the goal was to identify systems that are employed to determine credential equivalency, with a focus on Canada. Although Covell et al. [10] completed a scoping review, the focus was to identify motivation themes for the integration of internationally educated health professionals into the Canadian healthcare system. The goal of this review was to: (1) synthesize all the literature related to policies that impact immigration into Canada; (2) determine the *system*(s) that is/are employed to establish international equivalency of a professional with the ultimate goal of immigration and professional practice in a country that is different than their original domestic education [10]; and (3) include a wider definition beyond the healthcare workforce, since equivalency systems from other non-healthcare-related professions may be applicable across multiple disciplines.

## Methods

A scoping review method was chosen, because much of the data we are searching for can be found on professional governing body websites, whereas the traditional academic literature is less well-known. A scoping review was carried out by employing a six-stage methodological framework [10, 11]. The methods and study protocol were registered in the Open Science Framework (https://doi.org/10.17605/OSF.IO/3XD45).

### Stage 1: Identifying the research questions

The research question is to search and synthesize all the literature related to *system*(s) that is/are employed to establish international credential equivalency of a professional with the ultimate goal of immigration and professional practice in a country that is different than their original domestic education. To provide more context to the research question, systems will be classified as: 1 = paper; 2 = electronic; 3 = combination of both paper and electronic; 4 = machine-learning or some form of automation. The other key frame of reference for the research question of this scoping review is related the definition of a profession. Therefore, the following general framework and definition for “profession” was adapted from Luthans as cited in Marutello [12].

1. A body of specialized knowledge or techniques
2. Formal, standardized education, training and experience.
3. A representative organization with the purpose of professionalization.
4. Fees based on services to clients or customers with priority given to service rather than financial return.
5. An ethical code of conduct and broad-based responsibility.

Twenty professional groups that met these criteria were selected for this study (Table 1). Despite a focus for this review being “healthcare-related,” four non-healthcare-related professions (#17–20 inclusively) were also added to the search strategy with the goal to identify exemplars of international credential equivalency systems.

**Table 1:** List of professions included in search strategy

Number	Profession
1	Medicine
2	Nursing
3	Athletic training/therapy
4	Physiotherapy
5	Occupational therapy
6	Chiropractic
7	Dentistry
8	Dental hygiene
9	Paramedics (pre-hospital care)
10	Dietician
11	X-ray technicians
12	Psychologists
13	Massage therapy
14	Pharmacist
15	Kinesiologist
16	Respiratory therapist
17	Teachers
18	Accountant
19	Law
20	Engineering

### Stage 2: Identifying the academic and grey literature

Seven databases were searched to identify relevant sources: MEDLINE, CINAHL Plus with Full Text, Academic Search Complete, PsycINFO, SPORT Discus, Business Source Complete, and SCOPUS. No date limitations were set; therefore, the searches covered the time period from database inception to January 2022. The search strategy combined keyword, text terms, and medical subject headings (MeSH) and was carried out with the help of a health sciences librarian. The full search strategy is presented in Additional file 1. The bibliographies of selected articles and relevant systematic reviews were hand searched to identify additional articles not identified by the search strategy. Only English language studies were included. Editorials were also excluded.

### Stage 3: Selecting the literature

Two authors (ML and UM) were responsible for independently evaluating all titles corresponding abstracts retrieved from the literature search. After a full title and abstract review, data were compiled and consensus was reached for disagreements between the two reviewers regarding potentially relevant articles. Full-text articles of potentially relevant articles were reviewed and literature on competency frameworks, competencies, milestones, entrustable professional activities, certification/licensure standards, program accreditation standards, clinical education standards, and other practical skill expectations were included. ML and UM also reviewed the websites and grey literature of professions reported in Table 1.

### Stage 4: Extracting and charting the data

Data were extracted from all literature (i.e., article, website, grey literature) included: authors, publication year, profession, system name, and criteria used in the evaluation. Reference to a *system* was primary exclusion criteria that was used to extract the data. Both authors reviewed respective credential equivalency recognition systems and criteria used in the evaluation process. 

### Stage 5: Collating, summarizing and reporting the results

Once again, literature that referenced systems were categorized into: 1 = paper; 2 = electronic; 3 = combination of both paper and electronic; 4 = machine-learning. In addition, the “grey literature” or in our case, the professional governing body websites that referenced systems employed to determine international equivalency also used the same data categorization system.

### Stage 6: Consultation

The authors of this paper acted as the advisory group to guide the research question, inclusion and exclusion criteria and, where necessary, tie-breaking for inclusion or exclusion of data or literature sources of data.

## Results

**Fig. 1:**
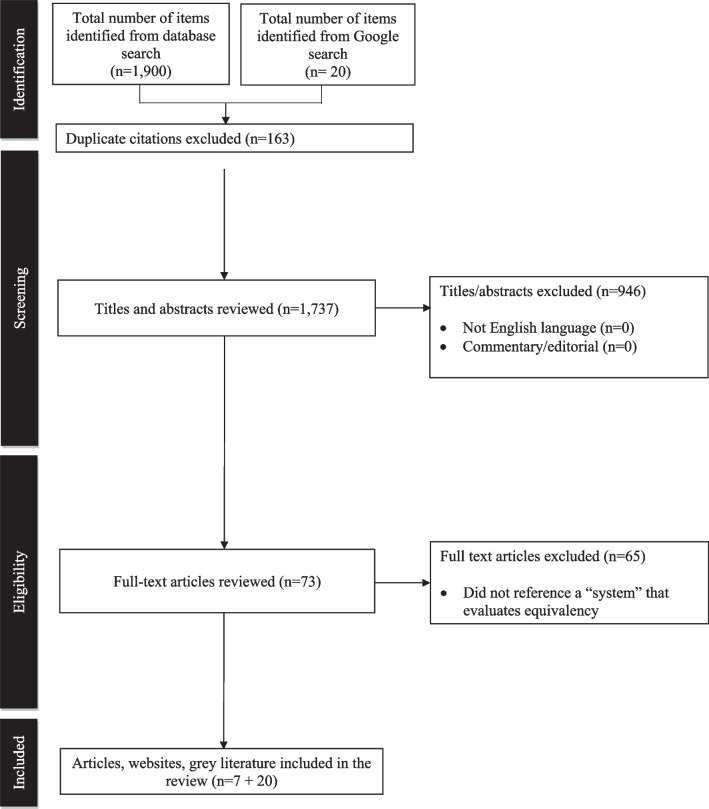
Search strategy flow chart results

The search of six databases identified 1900 articles that met the search criteria initially (Fig. 1). There were seven papers that met the criteria for full text review after removing 163 duplicates and screening by two authors (ML and UM).

The seven articles that were included in the final manuscript review were from the following professions: nursing [13, 14]; psychology [12, 13]; engineering [17]; pharmacy [18]; and multiple health professions [19]. Five of the seven papers were Canadian-based professions and the other two papers were from Asia or America. Equivalency criteria that were extracted from the seven papers and are listed in Table 2.

**Table 2:** Results from the scoping review including a summary of the system employed and criteria used in equivalency evaluation

Paper #	Discipline referenced	Systems employed*	Equivalency criteria**
1. Kwan et al., 2019 [13]	Nursing	3	1, 2, 3, 6, 7, 13, 14, 23
2. Mercier et al., 2021 [14]	Nursing	Specific type was not reported	11, 13, 19, 22
3. Gauthier et al., 2002[15]	Psychology	Specific type was not reported	3, 11, 14, 21, 24,
4. Rodolfa et al., 2005[16]	Psychology	4	3, 13, 17, 21, 24,
5. Hamanaka et al., 2021[17]	Engineering	3	2, 3, 6, 11, 13, 14, 21
6. Austin et al., 2007[18]	Pharmacy	4	1, 3, 4, 8, 9, 13, 20, 22, 23,
7. Augustine et al., 2015 [19]^	Accounting, Engineering and Medicine to name 3/38 referenced	n/a—mostly referenced systems, but were not specific	13, 22

^*^Where 1 = paper; 2 = electronic; 3 = combination of both paper and electronic; 4) competency-based; 5 = machine-learning system

^**^The numbers associated with the equivalency criteria legend are listed in Table 3

^This study focused on cross border equivalency between Canadian provinces primarily

## Manual searching of professional governing bodies websites in Canada

The manual searching of the Canadian professional governing bodies (Table 1) websites and grey literature revealed a number a variety of systems to evaluate international equivalency. Those criteria are listed in Table 3 broken down by each professional governing body.

**Table 3:** Canadian professional governing bodies international equivalency systems extracted from their websites

Number	Profession	Systems employed*	Equivalency criteria**
1	Medicine	BC, AB, ON 2 MB, QC 3	BC 1, 2, 4, 14, 15 AB 1, 3, 4, 8, 14 MB 2, 4, 10, 14–16 ON 3, 4 QC 1, 3, 5, 14
2	Nursing	BC, AB, MB, ON 3 QC 2	BC 1, 3, 5, 6, 13, 18 AB 1, 3, 5, 6, 13, 16, 18 MB 1, 3, 5, 6, 13, 18 ON 1, 2, 3, 5, 6, 13, 18 QC1, 5, 6, 13–15, 18, 20
3	Athletic training/therapy	2	3, 5, 6, 7
4	Physiotherapy	AB, MB 1 BC, ON, QC 3	BC 1, 3, 6, 9, 11, 13, 19 AB 1, 3, 6, 9, 11, 13 MB1, 3, 6, 9, 11, 13, 18 ON 1, 3, 5, 6, 9, 11, 13 QC 3–6, 8, 11, 13, 15, 18
5	Occupational therapy	1 or 2	1, 6
6	Chiropractic	BC, AB, MB, ON 2 QC 3	BC 3, 5, 6 AB—unknown MB 3, 5, 6 ON 1, 3, 16 QC 3, 8, 11, 13, 14
7	Dentistry	3	From accredited program—3, 4, 5, 6, 13, 16, 25–27 From non-accredited program—5, 6, 9, 13, 27
8	Dental hygiene	3	1, 3, 6, 8, 16
9	Paramedics (pre-hospital care)	BC, AB, MB, ON 2 QC 3	BC, AB, MB, ON 1, 2, 5, 8, 10, 13, 15, 18 QC 13, 25, 28, 29
10	Dietician	3	1, 2, 5, 6, 8, 9, 10, 11
11	X-ray technicians	1	1, 2, 5, 6, 8, 10, 13, 14, 15, 16
12	Psychologists	2	1, 3, 6, 8
13	Massage therapy	3	1, 3, 6, 8, 10, 13, 15, 16
14	Pharmacist	3	1, 5, 6, 8
15	Kinesiologist	BC 2 QC, ON 3	BC 1, 4, 6, 8, 16 ON 1, 3, 5, 6, 8 QC 6, 13 Other provinces 1, 6, 8
16	Respiratory therapist	3	1, 4, 5, 6, 11, 13, 16
17	Teachers	3	1, 2, 5, 6, 7
18	Accountant	3	MRA Ireland 3, 5, 6, 12–15 MRA Mexico and US 3, 5, 11–15 RMA 5, 12–15 MOU India and Pakistan 11 Not part of agreement 1, 5, 6, 8, 11, 13
19	Law	3	1, 3, 5, 6, 15, 17
20	Engineering	3	1, 14, 16

^*^Where 1 = paper; 2 = electronic; 3 = combination of both paper and electronic; 4 = competency-based; 5 = machine-learning system

^**^The numbers associated with the equivalency criteria legend are listed in Table 4

Criteria used to determine international equivalency were extracted from both the seven articles that were part of the scoping review as well as the professional governing body websites (Table 4).

**Table 4:** Criteria used to determine international equivalency

Criteria used to evaluate equivalency	
English language	1
Currency of practice	2
Other exams or certifications	3
Postgraduate training/courses	4
Statement of standing/professional memberships	5
Post-secondary transcripts	6
Secondary school leaving certificate	7
Academic course description/outline/syllabus	8
Internship verification letter/preceptorship	9
Self-assessment form to integrated competencies/self-assessment of practice/personal statement	10
Practical experience/practice hours	11
Pre-designation international employment	12
Post-secondary education degrees/diplomas/academic qualification	13
Work experience	14
Resume	15
Evidence of good character/reference letter	16
Research requirement	17
Employer letter of offer/employment certificate	18
Regulatory history form	19
Professional integration program	20
Competency assessment/comparison	21
Domestic cultural experience	22
Scope of practice	23
Accreditation standards for academic programs	24

## Discussion

One common theme among professions in Canada is that their governance and policies are aimed at protecting the public through standardization of professional standards, including education, program accreditation and certification to name a few [16]. Those same expectations must be maintained even with internationally education and credentialing received abroad. Canada has a long history of immigration with a particular focus on targeting and integrating foreign-trained healthcare [18, 20]. In fact, the Canadian Ministry of Employment and Social Development has dedicated tremendous resources to a foreign credential recognition program [21]. Professions such as pharmacy [18] or nursing [13] have a particular focus or interest in credential recognition in the hopes of meeting the policy and immigration demands in Canada. However, these professions have run into a number of barriers to credential recognition process, including establishing equivalency between nations or even between provinces. In addition, a substantial proportion of Canadian citizens who receive international training encounter significant barriers when trying to return home to practice, and end up moving to the United States, Britain, and Australia.

This scoping review aimed to uncover the various systems that are employed by many professions both nationally and internationally. Secondarily, this scoping review also aimed to determine the criteria that are employed within systems to make decisions about credential recognition and equivalency.

The scoping review revealed that most systems use a competency assessment that were driven by paper and/or electronic methods as their system of evaluation. It was surmised that most evaluation processes are still completed manually by individuals with expertise in the criteria their profession has required to determine equivalency. It should be noted that the scoping review did not find any profession or professional organization to employ artificial intelligence.

Existing systems for competency evaluation require human intervention to interpret the data and make the equivalency decisions. Relying solely on human interpretation can be time-consuming labor-intensive and, the quality of any labor-intensive process can be affected by human oversight. Computational algorithms and automated decision support systems (DSS) may enhance this manual process. DSSs have shown tremendous success in automating processes and supporting human decisions in different domains, such as in higher education, health care [22], education [23], agriculture [24, 25], and finance [26]. These DSSs benefit from artificial intelligence and machine learning techniques that can identify patterns and use them in their decision-making process. Future research should focus on the use of automated DSSs in the competency equivalency evaluation domain, since it has not been explored previously.

The criteria for determining international equivalency for health-related professions as outlined in Table 4 need be extracted from various textual documents, such as resume, reference letters, and educational records submitted by applicants. These documents are in the form of unstructured or free text data. Text and natural language processing (NLP) techniques can be used to extract useful data from these documents, to understand the sentiment of a text or to summarize long documents. For example, sentiment analysis [27] can be used to automatically highlight the evidence of good character/reference letter and speed up the equivalency decision process. NLP techniques have been used successfully in different applications, such as recruitment processes [26–28], document verification [28] education [29], and health science [30]. Yang and Heines [31] have employed text processing and semantic distances among words, sentences, and paragraphs for course transfer equivalencies between universities and show the potential of using these techniques in more complex competency evaluation. International equivalency and competency evaluation requires analysis of different types of documents and hence requires more advanced processes and algorithms to be designed.

### Limitations

There are a number of limitations to this study. The first and most obvious are the parameters and words that were chosen to search the databases for systems that are employed to establish international equivalency between countries or jurisdictions. The second most obvious limitation is that fact that we were only looking for “systems” as a key variable when reading the manuscripts that came from the formal search.

## Conclusions and future research

Many systems are used to establish international equivalency across health and non-health-related professions. However, this scoping review revealed there were no systems employ any form of artificial intelligence or machine learning to automate the decision-making process. Further research is required to design algorithms for competency evaluation. The 24 criteria that were unearthed in this scoping review process can be used as a foundation. Rule-based techniques can be designed to extract useful information from the documents or to summarize long documents using text processing techniques to speed up the evaluation process.

### Supplementary Information

**Additional file 1.** Search Strategies for the Medline, PsychoInfo, Sport Discus, Academic Search Complete/CINAHL, Business Complete and Scopus.

## Data Availability

If published, we will make our data available. However, saying that, we have included our search string and results of those search strings in the manuscript and appendix.
