# Correction: Systems that evaluate international equivalency in health-related professions: a scoping review with a focus on Canada

**DOI:** 10.1186/s12960-024-00894-0

**Published:** 2024-01-31

**Authors:** Mark Lafave, Yasaman Amannejad, Ulkar Mammadova, Breda Eubank

**Affiliations:** Mount Royal University, 4825 Mount Royal Gate SW, Calgary, AB T3E 6K6 Canada

**Correction: Human Resources for Health (2023) 21:79** 10.1186/s12960-023-00864-y

Following publication of the original article [1], the authors identified an error in the author name of Yasaman Amannejad.

The incorrect author name is: Yasaman Ammanejad.

The correct author name is: Yasaman Amannejad.

The author group has been updated above and the original article [1] has been corrected.

## References

[1] Lafave M, Amannejad Y, Mammadova U, Eubank B (2023). Systems that evaluate international equivalency in health-related professions: a scoping review with a focus on Canada. Hum Resour Health.

