# Supplementary material for: Systems that evaluate international equivalency in health-related professions: a scoping review with a focus on Canada
Source: Hum Resour Health. 2023 Oct 6;21:79. doi: 10.1186/s12960-023-00864-y (PMC10559399; doi:10.1186/s12960-023-00864-y)
Supplement: Supplementary file 1 — Additional file 1. Search Strategies for the Medline, PsychoInfo, Sport Discus, Academic Search Complete/CINAHL, Business Complete and Scopus. [file 12960_2023_864_MOESM1_ESM.docx]

**Additional file 1**

**Search Strategies for the Medline, PsychoInfo, Sport Discus, Academic Search Complete/CINAHL, Business Complete and Scopus**

Database(s): **Ovid MEDLINE(R) ALL**1946 to February 03, 2023
Search Strategy:

| **#** | **Searches** | **Results** |
| --- | --- | --- |
| 1 | exp Professional Competence/ | 128372 |
| 2 | exp Clinical Competence/ | 104139 |
| 3 | exp Credentialing/ | 57482 |
| 4 | exp Certification/ | 19287 |
| 5 | exp Licensure/ | 18239 |
| 6 | exp Accreditation/ | 19884 |
| 7 | exp Internationality/ | 176458 |
| 8 | exp "Joint Commission on Accreditation of Healthcare Organizations"/ | 7465 |
| 9 | exp "Emigration and Immigration"/ | 26188 |
| 10 | Mutual recognition agreement.mp. | 13 |
| 11 | equivalency.mp. | 11118 |
| 12 | interchangeability.mp. | 1833 |
| 13 | internationally educated health professionals.mp. | 17 |
| 14 | internationally educated health care professionals.mp. | 2 |
| 15 | health worker recruitment.mp. | 17 |
| 16 | 1 or 2 or 3 or 4 or 5 or 6 or 7 or 8 or 9 | 368997 |
| 17 | 10 or 11 or 12 or 13 or 14 or 15 | 12834 |
| 18 | 16 and 17 | 304 |
| 19 | limit 18 to english language | 277 |

Database(s): **APA PsycInfo**1806 to January Week 4 2023
Search Strategy:

| **#** | **Searches** | **Results** |
| --- | --- | --- |
| 1 | exp Professional Competence/ | 13367 |
| 2 | exp Competence/ | 30376 |
| 3 | exp Clinical Practice/ | 23594 |
| 4 | exp Professional Certification/ | 2476 |
| 5 | exp Professional Licensing/ | 1772 |
| 6 | exp Professional Standards/ | 9628 |
| 7 | exp Educational Program Accreditation/ | 836 |
| 8 | exp Professional Examinations/ | 1091 |
| 9 | exp "Accreditation (Education Personnel)"/ | 308 |
| 10 | exp International Relations/ | 3651 |
| 11 | exp Globalization/ | 10240 |
| 12 | Mutual recognition agreement.mp. | 5 |
| 13 | equivalency.mp. | 1047 |
| 14 | interchangeability.mp. | 289 |
| 15 | internationally educated health professionals.mp. | 7 |
| 16 | internationally educated health care professionals.mp. | 2 |
| 17 | 1 or 2 or 3 or 4 or 5 or 6 or 7 or 8 or 9 or 10 or 11 | 79858 |
| 18 | 12 or 13 or 14 or 15 or 16 | 1347 |
| 19 | 17 and 18 | 30 |
| 20 | limit 19 to english language | 30 |

Database(s): **SPORTDiscus with Full Text**February 6, 2023
Search Strategy:

| **#** | **Query** | **Limiters/Expanders** | **Last Run Via** | **Results** |
| --- | --- | --- | --- | --- |
| S27 | S25 AND S26 | Expanders - Apply equivalent subjects  Search modes – Find any of my search terms | Interface - EBSCOhost Research Databases  Search Screen - Advanced Search  Database – SPORTDiscus with Full-Text | 120 |
| S26 | S19 OR S20 OR S21 OR S22 OR S23 OR S24 | Expanders - Apply equivalent subjects  Search modes – Find any of my search terms | Interface - EBSCOhost Research Databases  Search Screen - Advanced Search  Database – SPORTDiscus with Full-Text | 1,008 |
| S25 | S1 OR S2 OR S3 OR S4 OR S5 OR S6 OR S7 OR S8 OR S9 OR S10 OR S11 OR S12 OR S13 OR S14 OR S15 OR S16 OR S17 OR S18 | Expanders - Apply equivalent subjects  Search modes – Find any of my search terms | Interface - EBSCOhost Research Databases  Search Screen - Advanced Search  Database – SPORTDiscus with Full-Text | 80,805 |
| S24 | Athletic training license | Limiters - English Language; Human  Expanders - Apply equivalent subjects  Search modes - Boolean/Phrase | Interface - EBSCOhost Research Databases  Search Screen - Advanced Search  Database – SPORTDiscus with Full-Text | 1 |
| S23 | Internationally educated health care professionals | Limiters - English Language; Human  Expanders - Apply equivalent subjects  Search modes - Boolean/Phrase | Interface - EBSCOhost Research Databases  Search Screen - Advanced Search  Database – SPORTDiscus with Full-Text | 665 |
| S22 | Internationally educated health professionals | Limiters - English Language; Human  Expanders - Apply equivalent subjects  Search modes - Boolean/Phrase | Interface - EBSCOhost Research Databases  Search Screen - Advanced Search  Database – SPORTDiscus with Full-Text | 631 |
| S21 | Interchangeability | Limiters - English Language; Human  Expanders - Apply equivalent subjects  Search modes - Boolean/Phrase | Interface - EBSCOhost Research Databases  Search Screen - Advanced Search  Database – SPORTDiscus with Full-Text | 76 |
| S20 | Equivalence | Limiters - English Language; Human  Expanders - Apply equivalent subjects  Search modes - Boolean/Phrase | Interface - EBSCOhost Research Databases  Search Screen - Advanced Search  Database – SPORTDiscus with Full-Text | 890 |
| S19 | Mutual recognition agreement | Limiters - English Language; Human  Expanders - Apply equivalent subjects  Search modes - Boolean/Phrase | Interface - EBSCOhost Research Databases  Search Screen - Advanced Search  Database – SPORTDiscus with Full-Text | 4 |
| S18 | Certification | Limiters - English Language; Human  Expanders - Apply equivalent subjects  Search modes - Boolean/Phrase | Interface - EBSCOhost Research Databases  Search Screen - Advanced Search  Database – SPORTDiscus with Full-Text | 9,108 |
| S17 | Globalization | Limiters - English Language; Human  Expanders - Apply equivalent subjects  Search modes - Boolean/Phrase | Interface - EBSCOhost Research Databases  Search Screen - Advanced Search  Database – SPORTDiscus with Full-Text | 2,012 |
| S16 | National competency-based educational tests | Limiters - English Language; Human  Expanders - Apply equivalent subjects  Search modes - Boolean/Phrase | Interface - EBSCOhost Research Databases  Search Screen - Advanced Search  Database – SPORTDiscus with Full-Text | 185 |
| S15 | Interdisciplinary education | Limiters - English Language; Human  Expanders - Apply equivalent subjects  Search modes - Boolean/Phrase | Interface - EBSCOhost Research Databases  Search Screen - Advanced Search  Database – SPORTDiscus with Full-Text | 925 |
| S14 | Educational accreditation | Limiters - English Language; Human  Expanders - Apply equivalent subjects  Search modes - Boolean/Phrase | Interface - EBSCOhost Research Databases  Search Screen - Advanced Search  Database – SPORTDiscus with Full-Text | 216 |
| S13 | License agreements | Limiters - English Language; Human  Expanders - Apply equivalent subjects  Search modes - Boolean/Phrase | Interface - EBSCOhost Research Databases  Search Screen - Advanced Search  Database – SPORTDiscus with Full-Text | 356 |
| S12 | Professional --law & legislation | Limiters - English Language; Human  Expanders - Apply equivalent subjects  Search modes - Boolean/Phrase | Interface - EBSCOhost Research Databases  Search Screen - Advanced Search  Database – SPORTDiscus with Full-Text | 36 |
| S11 | Licenses | Limiters - English Language; Human  Expanders - Apply equivalent subjects  Search modes - Boolean/Phrase | Interface - EBSCOhost Research Databases  Search Screen - Advanced Search  Database – SPORTDiscus with Full-Text | 3,748 |
| S10 | Professional standards | Limiters - English Language; Human  Expanders - Apply equivalent subjects  Search modes - Boolean/Phrase | Interface - EBSCOhost Research Databases  Search Screen - Advanced Search  Database – SPORTDiscus with Full-Text | 823 |
| S9 | Professional licensure examinations | Limiters - English Language; Human  Expanders - Apply equivalent subjects  Search modes - Boolean/Phrase | Interface - EBSCOhost Research Databases  Search Screen - Advanced Search  Database – SPORTDiscus with Full-Text | 108 |
| S8 | Standards | Expanders - Apply equivalent subjects  Search modes - Boolean/Phrase | Interface - EBSCOhost Research Databases  Search Screen - Advanced Search  Database – SPORTDiscus with Full-Text | 53,907 |
| S7 | Professional licenses | Expanders - Apply equivalent subjects  Search modes - Boolean/Phrase | Interface - EBSCOhost Research Databases  Search Screen - Advanced Search  Database – SPORTDiscus with Full-Text | 419 |
| S6 | Professional education | Expanders - Apply equivalent subjects  Search modes - Boolean/Phrase | Interface - EBSCOhost Research Databases  Search Screen - Advanced Search  Database – SPORTDiscus with Full-Text | 5,559 |
| S5 | Interprofessional relations | Expanders - Apply equivalent subjects  Search modes - Boolean/Phrase | Interface - EBSCOhost Research Databases  Search Screen - Advanced Search  Database – SPORTDiscus with Full-Text | 1,825 |
| S4 | Accreditation | Expanders - Apply equivalent subjects  Search modes - Boolean/Phrase | Interface - EBSCOhost Research Databases  Search Screen - Advanced Search  Database – SPORTDiscus with Full-Text | 2,306 |
| S3 | Cultural competence | Expanders - Apply equivalent subjects  Search modes - Boolean/Phrase | Interface - EBSCOhost Research Databases  Search Screen - Advanced Search  Database – SPORTDiscus with Full-Text | 464 |
| S2 | Professional practice | Expanders - Apply equivalent subjects  Search modes - Boolean/Phrase | Interface - EBSCOhost Research Databases  Search Screen - Advanced Search  Database – SPORTDiscus with Full-Text | 4,411 |
| S1 | Clinical competence | Expanders - Apply equivalent subjects  Search modes - Boolean/Phrase | Interface - EBSCOhost Research Databases  Search Screen - Advanced Search  Database – SPORTDiscus with Full-Text | 1,120 |

Database(s): **Academic Search Complete** February 6, 2023
Search Strategy:

| **#** | **Query** | **Limiters/Expanders** | **Last Run Via** | **Results** |
| --- | --- | --- | --- | --- |
| S25 | S23 AND S24 | Expanders - Apply equivalent subjects  Search modes – Find any of my search terms | Interface - EBSCOhost Research Databases  Search Screen - Advanced Search  Database – Academic Search Complete | 262 |
| S24 | S19 OR S20 OR S21 ORS22 | Expanders - Apply equivalent subjects  Search modes – Find any of my search terms | Interface - EBSCOhost Research Databases  Search Screen - Advanced Search  Database – Academic Search Complete | 1,626 |
| S23 | S1 OR S2 OR S3 OR S4 OR S5 OR S6 OR S7 OR S8 OR S9 OR S10 OR S11 OR S12 OR S13 OR S14 OR S15 OR S16 OR S17 OR S18 | Expanders - Apply equivalent subjects  Search modes – Find any of my search terms | Interface - EBSCOhost Research Databases  Search Screen - Advanced Search  Database – Academic Search Complete | 1,580,689 |
| S22 | Internationally educated professionals | Limiters - English Language; Human  Expanders - Apply equivalent subjects  Search modes - Boolean/Phrase | Interface - EBSCOhost Research Databases  Search Screen - Advanced Search  Database – Academic Search Complete | 22 |
| S21 | Interchangeability | Limiters - English Language; Human  Expanders - Apply equivalent subjects  Search modes - Boolean/Phrase | Interface - EBSCOhost Research Databases  Search Screen - Advanced Search  Database – Academic Search Complete | 1,453 |
| S20 | Professional equivalenc* | Limiters - English Language; Human  Expanders - Apply equivalent subjects  Search modes - Boolean/Phrase | Interface - EBSCOhost Research Databases  Search Screen - Advanced Search  Database – Academic Search Complete | 38 |
| S19 | Mutual recognition agreement | Limiters - English Language; Human  Expanders - Apply equivalent subjects  Search modes - Boolean/Phrase | Interface - EBSCOhost Research Databases  Search Screen - Advanced Search  Database – Academic Search Complete | 113 |
| S18 | Certification | Limiters - English Language; Human  Expanders - Apply equivalent subjects  Search modes - Boolean/Phrase | Interface - EBSCOhost Research Databases  Search Screen - Advanced Search  Database – Academic Search Complete | 47,663 |
| S17 | Globalization | Limiters - English Language; Human  Expanders - Apply equivalent subjects  Search modes - Boolean/Phrase | Interface - EBSCOhost Research Databases  Search Screen - Advanced Search  Database – Academic Search Complete | 69,530 |
| S16 | National competency-based educational tests | Limiters - English Language; Human  Expanders - Apply equivalent subjects  Search modes - Boolean/Phrase | Interface - EBSCOhost Research Databases  Search Screen - Advanced Search  Database – Academic Search Complete | 2,347 |
| S15 | Interdisciplinary education | Limiters - English Language; Human  Expanders - Apply equivalent subjects  Search modes - Boolean/Phrase | Interface - EBSCOhost Research Databases  Search Screen - Advanced Search  Database – Academic Search Complete | 9,989 |
| S14 | Educational accreditation | Limiters - English Language; Human  Expanders - Apply equivalent subjects  Search modes - Boolean/Phrase | Interface - EBSCOhost Research Databases  Search Screen - Advanced Search  Database – Academic Search Complete | 6,601 |
| S13 | License agreements | Limiters - English Language; Human  Expanders - Apply equivalent subjects  Search modes - Boolean/Phrase | Interface - EBSCOhost Research Databases  Search Screen - Advanced Search  Database – Academic Search Complete | 6,153 |
| S12 | Professional --law & legislation | Limiters - English Language; Human  Expanders - Apply equivalent subjects  Search modes - Boolean/Phrase | Interface - EBSCOhost Research Databases  Search Screen - Advanced Search  Database – Academic Search Complete | 137 |
| S11 | Licenses | Limiters - English Language; Human  Expanders - Apply equivalent subjects  Search modes - Boolean/Phrase | Interface - EBSCOhost Research Databases  Search Screen - Advanced Search  Database – Academic Search Complete | 52,316 |
| S10 | Professional standards | Limiters - English Language; Human  Expanders - Apply equivalent subjects  Search modes - Boolean/Phrase | Interface - EBSCOhost Research Databases  Search Screen - Advanced Search  Database – Academic Search Complete | 8,970 |
| S9 | Professional licensure examinations | Limiters - English Language; Human  Expanders - Apply equivalent subjects  Search modes - Boolean/Phrase | Interface - EBSCOhost Research Databases  Search Screen - Advanced Search  Database – Academic Search Complete | 801 |
| S8 | Standards | Expanders - Apply equivalent subjects  Search modes - Boolean/Phrase | Interface - EBSCOhost Research Databases  Search Screen - Advanced Search  Database – Academic Search Complete | 1,297,141 |
| S7 | Professional licenses | Expanders - Apply equivalent subjects  Search modes - Boolean/Phrase | Interface - EBSCOhost Research Databases  Search Screen - Advanced Search  Database – Academic Search Complete | 2,127 |
| S6 | Professional education | Expanders - Apply equivalent subjects  Search modes - Boolean/Phrase | Interface - EBSCOhost Research Databases  Search Screen - Advanced Search  Database – Academic Search Complete | 42,570 |
| S5 | Interprofessional relations | Expanders - Apply equivalent subjects  Search modes - Boolean/Phrase | Interface - EBSCOhost Research Databases  Search Screen - Advanced Search  Database – Academic Search Complete | 22,062 |
| S4 | Accreditation | Expanders - Apply equivalent subjects  Search modes - Boolean/Phrase | Interface - EBSCOhost Research Databases  Search Screen - Advanced Search  Database – Academic Search Complete | 24,293 |
| S3 | Cultural competence | Expanders - Apply equivalent subjects  Search modes - Boolean/Phrase | Interface - EBSCOhost Research Databases  Search Screen - Advanced Search  Database – Academic Search Complete | 7,788 |
| S2 | Professional practice | Expanders - Apply equivalent subjects  Search modes - Boolean/Phrase | Interface - EBSCOhost Research Databases  Search Screen - Advanced Search  Database – Academic Search Complete | 42,584 |
| S1 | Clinical competence | Expanders - Apply equivalent subjects  Search modes - Boolean/Phrase | Interface - EBSCOhost Research Databases  Search Screen - Advanced Search  Database – Academic Search Complete | 16,019 |

Database(s): **Business Source Complete** February 6, 2023
Search Strategy:

| **#** | **Query** | **Limiters/Expanders** | **Last Run Via** | **Results** |
| --- | --- | --- | --- | --- |
| S24 | S22 AND S23 | Expanders - Apply equivalent subjects  Search modes – Find any of my search terms | Interface - EBSCOhost Research Databases  Search Screen - Advanced Search  Database – Business Source Complete | 1,851 |
| S23 | S18 OR S19 OR S20 OR S21 | Expanders - Apply equivalent subjects  Search modes – Find any of my search terms | Interface - EBSCOhost Research Databases  Search Screen - Advanced Search  Database – Business Source Complete | 23,594 |
| S22 | S1 OR S2 OR S3 OR S4 OR S5 OR S6 OR S7 OR S8 OR S9 OR S10 OR S11 OR S12 OR S13 OR S14 OR S15 OR S16 OR S17 | Expanders - Apply equivalent subjects  Search modes – Find any of my search terms | Interface - EBSCOhost Research Databases  Search Screen - Advanced Search  Database – Business Source Complete | 816,356 |
| S21 | Internationally educated professionals | Limiters - English Language; Human  Expanders - Apply equivalent subjects  Search modes - Boolean/Phrase | Interface - EBSCOhost Research Databases  Search Screen - Advanced Search  Database – Business Source Complete | 12 |
| S20 | Interchangeability | Limiters - English Language; Human  Expanders - Apply equivalent subjects  Search modes - Boolean/Phrase | Interface - EBSCOhost Research Databases  Search Screen - Advanced Search  Database – Business Source Complete | 405 |
| S19 | Equivalence | Limiters - English Language; Human  Expanders - Apply equivalent subjects  Search modes - Boolean/Phrase | Interface - EBSCOhost Research Databases  Search Screen - Advanced Search  Database – Business Source Complete | 22,994 |
| S18 | Mutual recognition agreement | Limiters - English Language; Human  Expanders - Apply equivalent subjects  Search modes - Boolean/Phrase | Interface - EBSCOhost Research Databases  Search Screen - Advanced Search  Database – Business Source Complete | 195 |
| S17 | Globalization | Limiters - English Language; Human  Expanders - Apply equivalent subjects  Search modes - Boolean/Phrase | Interface - EBSCOhost Research Databases  Search Screen - Advanced Search  Database – Business Source Complete | 56,180 |
| S16 | National competency-based educational tests | Limiters - English Language; Human  Expanders - Apply equivalent subjects  Search modes - Boolean/Phrase | Interface - EBSCOhost Research Databases  Search Screen - Advanced Search  Database – Business Source Complete | 202 |
| S15 | Interdisciplinary education | Limiters - English Language; Human  Expanders - Apply equivalent subjects  Search modes - Boolean/Phrase | Interface - EBSCOhost Research Databases  Search Screen - Advanced Search  Database – Business Source Complete | 1,002 |
| S14 | Educational accreditation | Limiters - English Language; Human  Expanders - Apply equivalent subjects  Search modes - Boolean/Phrase | Interface - EBSCOhost Research Databases  Search Screen - Advanced Search  Database – Business Source Complete | 3,573 |
| S13 | License agreements | Limiters - English Language; Human  Expanders - Apply equivalent subjects  Search modes - Boolean/Phrase | Interface - EBSCOhost Research Databases  Search Screen - Advanced Search  Database – Business Source Complete | 18,667 |
| S12 | Professional --law & legislation | Limiters - English Language; Human  Expanders - Apply equivalent subjects  Search modes - Boolean/Phrase | Interface - EBSCOhost Research Databases  Search Screen - Advanced Search  Database – Business Source Complete | 53 |
| S11 | Licenses | Limiters - English Language; Human  Expanders - Apply equivalent subjects  Search modes - Boolean/Phrase | Interface - EBSCOhost Research Databases  Search Screen - Advanced Search  Database – Business Source Complete | 96,015 |
| S10 | Professional standards | Limiters - English Language; Human  Expanders - Apply equivalent subjects  Search modes - Boolean/Phrase | Interface - EBSCOhost Research Databases  Search Screen - Advanced Search  Database – Business Source Complete | 7,021 |
| S9 | Professional licensure examinations | Limiters - English Language; Human  Expanders - Apply equivalent subjects  Search modes - Boolean/Phrase | Interface - EBSCOhost Research Databases  Search Screen - Advanced Search  Database – Business Source Complete | 163 |
| S8 | Standards | Expanders - Apply equivalent subjects  Search modes - Boolean/Phrase | Interface - EBSCOhost Research Databases  Search Screen - Advanced Search  Database – Business Source Complete | 596,599 |
| S7 | Professional licenses | Expanders - Apply equivalent subjects  Search modes - Boolean/Phrase | Interface - EBSCOhost Research Databases  Search Screen - Advanced Search  Database – Business Source Complete | 1,286 |
| S6 | Professional education | Expanders - Apply equivalent subjects  Search modes - Boolean/Phrase | Interface - EBSCOhost Research Databases  Search Screen - Advanced Search  Database – Business Source Complete | 14,046 |
| S5 | Interprofessional relations | Expanders - Apply equivalent subjects  Search modes - Boolean/Phrase | Interface - EBSCOhost Research Databases  Search Screen - Advanced Search  Database – Business Source Complete | 3,695 |
| S4 | Accreditation | Expanders - Apply equivalent subjects  Search modes - Boolean/Phrase | Interface - EBSCOhost Research Databases  Search Screen - Advanced Search  Database – Business Source Complete | 13,626 |
| S3 | Cultural competence | Expanders - Apply equivalent subjects  Search modes - Boolean/Phrase | Interface - EBSCOhost Research Databases  Search Screen - Advanced Search  Database – Business Source Complete | 1,113 |
| S2 | Professional practice | Expanders - Apply equivalent subjects  Search modes - Boolean/Phrase | Interface - EBSCOhost Research Databases  Search Screen - Advanced Search  Database – Business Source Complete | 8,332 |
| S1 | Competence | Expanders - Apply equivalent subjects  Search modes - Boolean/Phrase | Interface - EBSCOhost Research Databases  Search Screen - Advanced Search  Database – Business Source Complete | 45,050 |

Database(s): **CINAHL Plus with Full Text** February 6, 2023
Search Strategy:

| **#** | **Query** | **Limiters/Expanders** | **Last Run Via** | **Results** |
| --- | --- | --- | --- | --- |
| S27 | S25 AND S26 | Expanders - Apply equivalent subjects  Search modes – Find any of my search terms | Interface - EBSCOhost Research Databases  Search Screen - Advanced Search  Database – CINAHL Plus with Full Text | 133 |
| S26 | S20 OR S21 OR S22 OR S23 OR S24 | Expanders - Apply equivalent subjects  Search modes – Find any of my search terms | Interface - EBSCOhost Research Databases  Search Screen - Advanced Search  Database – CINAHL Plus with Full Text | 468 |
| S25 | S1 OR S2 OR S3 OR S4 OR S5 OR S6 OR S7 OR S8 OR S9 OR S10 OR S11 OR S12 OR S13 OR S14 OR S15 OR S16 OR S17 OR S18 OR S19 | Expanders - Apply equivalent subjects  Search modes – Find any of my search terms | Interface - EBSCOhost Research Databases  Search Screen - Advanced Search  Database – CINAHL Plus with Full Text | 636,009 |
| S24 | Internationally educated health professionals | Limiters - English Language; Human  Expanders - Apply equivalent subjects  Search modes - Boolean/Phrase | Interface - EBSCOhost Research Databases  Search Screen - Advanced Search  Database – CINAHL Plus with Full Text | 18 |
| S23 | Internationally educated professionals | Limiters - English Language; Human  Expanders - Apply equivalent subjects  Search modes - Boolean/Phrase | Interface - EBSCOhost Research Databases  Search Screen - Advanced Search  Database – CINAHL Plus with Full Text | 30 |
| S22 | Interchangeability | Limiters - English Language; Human  Expanders - Apply equivalent subjects  Search modes - Boolean/Phrase | Interface - EBSCOhost Research Databases  Search Screen - Advanced Search  Database – CINAHL Plus with Full Text | 402 |
| S21 | Professional equivalenc* | Limiters - English Language; Human  Expanders - Apply equivalent subjects  Search modes - Boolean/Phrase | Interface - EBSCOhost Research Databases  Search Screen - Advanced Search  Database – CINAHL Plus with Full Text | 16 |
| S20 | Mutual recognition agreement | Limiters - English Language; Human  Expanders - Apply equivalent subjects  Search modes - Boolean/Phrase | Interface - EBSCOhost Research Databases  Search Screen - Advanced Search  Database – CINAHL Plus with Full Text | 20 |
| S19 | Certification | Limiters - English Language; Human  Expanders - Apply equivalent subjects  Search modes - Boolean/Phrase | Interface - EBSCOhost Research Databases  Search Screen - Advanced Search  Database – CINAHL Plus with Full Text | 30,467 |
| S18 | Globalization | Limiters - English Language; Human  Expanders - Apply equivalent subjects  Search modes - Boolean/Phrase | Interface - EBSCOhost Research Databases  Search Screen - Advanced Search  Database – CINAHL Plus with Full Text | 2,345 |
| S17 | National competency-based educational tests | Limiters - English Language; Human  Expanders - Apply equivalent subjects  Search modes - Boolean/Phrase | Interface - EBSCOhost Research Databases  Search Screen - Advanced Search  Database – CINAHL Plus with Full Text | 3,500 |
| S16 | Interdisciplinary education | Limiters - English Language; Human  Expanders - Apply equivalent subjects  Search modes - Boolean/Phrase | Interface - EBSCOhost Research Databases  Search Screen - Advanced Search  Database – CINAHL Plus with Full Text | 7,567 |
| S15 | Educational accreditation | Limiters - English Language; Human  Expanders - Apply equivalent subjects  Search modes - Boolean/Phrase | Interface - EBSCOhost Research Databases  Search Screen - Advanced Search  Database – CINAHL Plus with Full Text | 6,093 |
| S14 | License agreement | Limiters - English Language; Human  Expanders - Apply equivalent subjects  Search modes - Boolean/Phrase | Interface - EBSCOhost Research Databases  Search Screen - Advanced Search  Database – CINAHL Plus with Full Text | 59 |
| S13 | Professional --law & legislation | Limiters - English Language; Human  Expanders - Apply equivalent subjects  Search modes – SmartText searching | Interface - EBSCOhost Research Databases  Search Screen - Advanced Search  Database – CINAHL Plus with Full Text | 288 |
| S12 | Professional --law & legislation | Limiters - English Language; Human  Expanders - Apply equivalent subjects  Search modes - Boolean/Phrase | Interface - EBSCOhost Research Databases  Search Screen - Advanced Search  Database – CINAHL Plus with Full Text | 0 |
| S11 | Licenses | Limiters - English Language; Human  Expanders - Apply equivalent subjects  Search modes - Boolean/Phrase | Interface - EBSCOhost Research Databases  Search Screen - Advanced Search  Database – CINAHL Plus with Full Text | 5,514 |
| S10 | Professional standards | Limiters - English Language; Human  Expanders - Apply equivalent subjects  Search modes - Boolean/Phrase | Interface - EBSCOhost Research Databases  Search Screen - Advanced Search  Database – CINAHL Plus with Full Text | 9,332 |
| S9 | Professional licensure examinations | Limiters - English Language; Human  Expanders - Apply equivalent subjects  Search modes - Boolean/Phrase | Interface - EBSCOhost Research Databases  Search Screen - Advanced Search  Database – CINAHL Plus with Full Text | 1,893 |
| S8 | Standards | Expanders - Apply equivalent subjects  Search modes - Boolean/Phrase | Interface - EBSCOhost Research Databases  Search Screen - Advanced Search  Database – CINAHL Plus with Full Text | 451,366 |
| S7 | Professional licenses | Expanders - Apply equivalent subjects  Search modes - Boolean/Phrase | Interface - EBSCOhost Research Databases  Search Screen - Advanced Search  Database – CINAHL Plus with Full Text | 114 |
| S6 | Professional education | Expanders - Apply equivalent subjects  Search modes - Boolean/Phrase | Interface - EBSCOhost Research Databases  Search Screen - Advanced Search  Database – CINAHL Plus with Full Text | 13,780 |
| S5 | Interprofessional relations | Expanders - Apply equivalent subjects  Search modes - Boolean/Phrase | Interface - EBSCOhost Research Databases  Search Screen - Advanced Search  Database – CINAHL Plus with Full Text | 29,682 |
| S4 | Accreditation | Expanders - Apply equivalent subjects  Search modes - Boolean/Phrase | Interface - EBSCOhost Research Databases  Search Screen - Advanced Search  Database – CINAHL Plus with Full Text | 18,444 |
| S3 | Cultural competence | Expanders - Apply equivalent subjects  Search modes - Boolean/Phrase | Interface - EBSCOhost Research Databases  Search Screen - Advanced Search  Database – CINAHL Plus with Full Text | 12,422 |
| S2 | Professional practice | Expanders - Apply equivalent subjects  Search modes - Boolean/Phrase | Interface - EBSCOhost Research Databases  Search Screen - Advanced Search  Database – CINAHL Plus with Full Text | 65,914 |
| S1 | Clinical competence | Expanders - Apply equivalent subjects  Search modes - Boolean/Phrase | Interface - EBSCOhost Research Databases  Search Screen - Advanced Search  Database – CINAHL Plus with Full Text | 48,472 |

Database(s): **SCOPUS** February 6, 2023
Search Strategy:

| ( (TITLE-ABS-KEY ( "professional licensing" ) OR T(ITLE-ABS-KEY ( "professional standards" )) OR ( TITLE-ABS-KEY ( accreditation )) OR (TITLE-ABS-KEY ( certification )) OR (TITLE-ABS-KEY ( competenc* ) ) OR (TITLE-ABS-KEY ( equivalenc* )) OR (ALL ( "interprofessional education" ) )) AND (ALL ( "mutual recognition agreement" ) ) | 116 |
| --- | --- |
